# l-Carnitine Supplementation in Recovery after Exercise

**DOI:** 10.3390/nu10030349

**Published:** 2018-03-13

**Authors:** Roger Fielding, Linda Riede, James P. Lugo, Aouatef Bellamine

**Affiliations:** 1 Tufts University, 136 Harrison Avenue, Boston, MA 02111, USA; roger.Fielding@tufts.edu; 2 analyze & realize GmbH, Waldseeweg 6, 13467 Berlin, Germany; lriede@a-r.com; 3 Lonza Inc., 90 Boroline Road, Allendale, NJ 07401, USA; jim.lugo@lonza.com

**Keywords:** l-carnitine, exercise recovery, physical performance, muscle metabolism, aging

## Abstract

Given its pivotal role in fatty acid oxidation and energy metabolism, l-carnitine has been investigated as ergogenic aid for enhancing exercise capacity in the healthy athletic population. Early research indicates its beneficial effects on acute physical performance, such as increased maximum oxygen consumption and higher power output. Later studies point to the positive impact of dietary supplementation with l-carnitine on the recovery process after exercise. It is demonstrated that l-carnitine alleviates muscle injury and reduces markers of cellular damage and free radical formation accompanied by attenuation of muscle soreness. The supplementation-based increase in serum and muscle l-carnitine contents is suggested to enhance blood flow and oxygen supply to the muscle tissue via improved endothelial function thereby reducing hypoxia-induced cellular and biochemical disruptions. Studies in older adults further showed that l-carnitine intake can lead to increased muscle mass accompanied by a decrease in body weight and reduced physical and mental fatigue. Based on current animal studies, a role of l-carnitine in the prevention of age-associated muscle protein degradation and regulation of mitochondrial homeostasis is suggested.

## 1. Introduction

**Figure 1:**
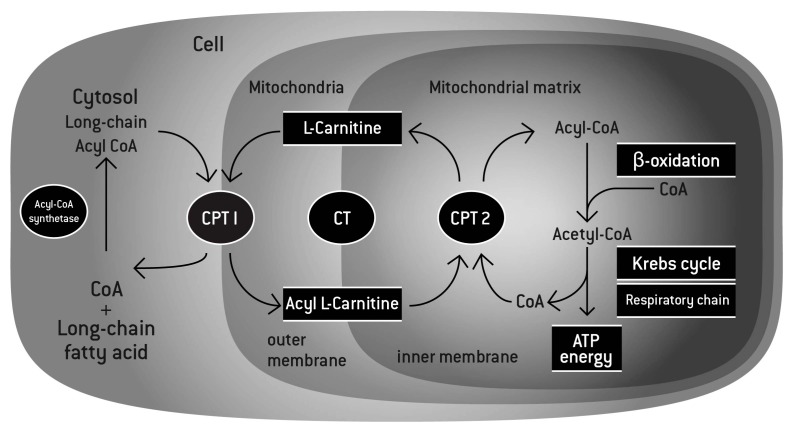
l-carnitine function. l-carnitine shuttles long-chain fatty acids inside the mitochondria by forming a long chain acetylcarnitine ester. The complex is then transported into the mitochondrial matrix by carnitine palmitoyltransferase I (CPT I) and carnitine palmitoyltransferase II (CPT II). The fatty acids are then broken down through the process of β-oxidation to deliver the 2-carbon molecules to the Krebs cycle, leading to the generation of energy under the form of adenosine triphosphate (ATP). In addition, by binding an acetyl group, l-carnitine can maintain the levels of Acetyl-CoA and coenzyme A, playing its buffering role.

Naturally occurring, l-carnitine is a quaternary amine (3-hydroxy-4-*N*-trimethylaminobutyrate) found in all mammalian species. After the discovery of l-carnitine in muscle extracts in 1905 [1] and its structural identification in 1927 [2], the importance of l-carnitine in fatty acid oxidation in the liver and the heart was first described by Fritz in 1959 [3]. As mitochondrial membranes are impermeable to coenzyme A (CoA) esters and long-chain fatty acids, binding of l-carnitine to acetyl groups via carnitine acyltransferase is essential for the shuttle of the acetylated fatty acids into the mitochondria and for their subsequent β-oxidation in the matrix (Figure 1) [4]. The products of the β-oxidation (two carbon molecules) are then used by the Krebs cycle to produce Adenosine triphosphate (ATP) as form of energy. l-carnitine has also been recognized for its crucial biological function in buffering the free CoA/acetyl-CoA ratio. Under conditions of stress with excess formation of acyl-CoA, transesterification with l-carnitine potentially promotes the substrate movement in the Krebs cycle.

l-carnitine is synthesized endogenously in the liver, the kidney, and the brain from the essential amino acids lysine and methionine [5,6] or ingested via animal-based food products. Its synthesis is catalyzed by four enzymatic reactions reviewed in detail by Vaz et al. [7] and requires vitamin C, vitamin B_6_, niacin, and reduced iron as cofactors [8]. Biosynthesis of l-carnitine accounts only for 25% of the daily needs [7,9]. Thus, supplementation either in the diet or as a nutritional supplement is required. At the tissue levels, the primary storage of l-carnitine is in the heart and the skeletal muscle with an estimated 95%, while much lower concentrations are found in the liver, the kidney, and the plasma [10]. It is estimated that the muscle content is about 70-fold higher than the blood plasma stores, which contain approximately 40–50 µM/L [11]. Therefore, uptake of l-carnitine depends on an energy-dependent transport system against the concentration gradient. The organic cation transporters (OCTNs) regulate tissue distribution and intracellular homeostasis of l-carnitine and function both on its intestinal absorption and renal reabsorption. Hereditary or acquired defects in the transport mechanisms are the major cause of l-carnitine deficiency, leading to pathologies such as cardiomyopathy and skeletal muscle myopathy [12,13].

It is estimated that, in omnivorous humans, 75% of the body’s carnitine pool is derived from dietary intake [7,9]. However, dietary intake of l-carnitine is highly variable. The primary source is red meat, providing up to 140–190 mg l-carnitine per 100 g uncooked meat (e.g., beef and venison) [14]. In contrast, plant-derived foods contain insignificant quantities of l-carnitine. As a consequence, vegetarians obtain very little l-carnitine from dietary sources. However, the benefit of l-carnitine supplementation in this population is still controversial as they possess comparable bioavailability for l-carnitine to the general population [12]. Indeed, it has been reported that l-carnitine deficit among vegetarians is modest [15]. With smaller l-carnitine stores in plasma as compared to omnivores, 12-weeks supplementation with l-carnitine increased both plasma and muscle carnitine, though muscle function and energy metabolism remained unaffected [16]. It is possible that regulatory feedback mechanisms leading to an increase in the dietary absorption of l-carnitine [17], and/or de novo synthesis [18] take place to overcome the l-carnitine deficiency and reduce the loss by urinary excretion. Such possible adaptation has been reported in vegetarians [16].

While the bioavailability of l-carnitine from dietary sources was estimated to be 54–86% [17], absorption of nutritional supplements was reported to be lower and to vary between doses with an uptake of 9–25% from a single oral dose of 2 g dose [17,19]. Part of the l-carnitine ingested can be metabolized by microbial species in the gut. It was demonstrated in animal studies that unabsorbed quaternary amines such as choline, phosphatidylcholine, betaine or l-carnitine can be metabolized by gut microorganisms to produce the intermediate compound trimethylamine (TMA). TMA is subsequently absorbed by the gut and oxidized by flavin-monooxygenases (FMOs) in the liver to produce trimethylamine-*N*-oxide (TMAO) [20]. However, these conversions depend on the microflora and the affinity of the different quaternary amines to the gut microbial populations. Recently, it was reported that conversion of choline into TMA is catalyzed by anaerobic bacteria whereas conversion from l-carnitine is an aerobic process, suggesting that l-carnitine is an inefficient source of TMA production. Similar process can take place in the gut [21].

Because of its accumulation in the muscle and the heart, its ergogenic nature, and its role in energy metabolism, l-carnitine supplementation is proposed to play crucial roles in diseased populations where it has been shown to impact the management of ischaemic heart disease, myopathy, and peripheral arterial disease [22,23,24], as well as among healthy athletes where it has been shown to modulate the exercise capacity and recovery [25,26,27,28].

This comprehensive review aims to summarize the role l-carnitine plays in muscle physiology with a focus on the recovery after exercise among athletes, describe some of the trials, and the potential mechanisms involved. Based on this learning and the proposed role of l-carnitine in muscle structure and function, the role of l-carnitine in muscle health during aging is also discussed.

## 2. Methodology

The literature search was done in the database “PubMed”. As a basic search string “carnitine AND exercise AND recovery” combined with the filter for human clinical trials was used. Based on the titles and abstracts, the relevance of the publications was judged. Excluded were trials were l-carnitine was given in combination with other products (multi-ingredient supplements) or the outcome was not related to post exercise recovery. Further publications written in languages other than English, German or French were excluded. Additional clinical trials were identified by carefully reading the publication list of the identified articles.

## 3. l-Carnitine and Exercise

The link between l-carnitine levels, particularly in the plasma and the muscle, and enhanced exercise capacity have been reported in many trials (Table 1). With the commercial availability of l-carnitine in the early 1980s, studies were initiated to examine the effect of l-carnitine supplementation on metabolism during exercise. In light of its fundamental role in the β-oxidation of fatty acids for the purpose of energy production, and its role in the regulation of the acetyl-CoA pool, studies on l-carnitine as an ergogenic aid initially focused on of its interaction with exercise. Arenas et al. first reported that dietary supplementation of 1 g of l-carnitine given twice daily during 6 months of exercise training led to an increase in muscle l-carnitine levels (total and free) compared to placebo [29]. Endurance runners and to a lesser extent sprinters showed a significant decrease in muscle free l-carnitine as a result of exercise only. These levels were reversed by l-carnitine supplementation [29]. In a different trial, Wachter et al., reported that supplementation with 2 g of l-carnitine given twice daily for a period of 3 months did not alter l-carnitine muscle levels [30]. However, in the trial, only post-exercise muscle biopsies have been collected at three months, which makes the assessment of chronic effects of the supplementation independently from exercise difficult. Indeed, exercise itself leads to l-carnitine depletion in the muscle [29]. In two different trials, Broad and his colleagues reported that supplementation with 2 or 3 g of l-carnitine for two weeks led to an improvement in glucose and ammonia plasma levels, as well as lowering in heart rate without affecting the fat and carbohydrate metabolisms [31,32]. It is possible that the observed effects are supported by others mechanisms than energy production. Such mechanisms will be detailed in the subsequent paragraph.

Other studies looked at the effect of supplementation on aerobic capacity, fat oxidation, maximum oxygen uptake, and physical performance [33,34,35,36,37,38]. Both chronic and acute supplementation with l-carnitine with or without protein during training, have been reported to enhance exercise capacity and endurance [33,34]. Orer and Guzel showed that single supplementation of 3 g or 4 g of l-carnitine to footballers before increasing speed run led to increased speed at corresponding lactate plasma levels, and decreased heart rate, suggesting a prolonged exhaustion exercise [36]. Siliprandi and colleagues provided evidence that l-carnitine enhances high-intensity exercise by maintaining the acetyl CoA/CoA ratio constant, thereby allowing continuous flux through the pyruvate dehydrogenase complex and preventing lactate accumulation [37]. Other studies yielded contradictory results, though, with outcomes unsupportive of a beneficial effect on maximal aerobic capacity, blood lactate response, or changes in respiratory exchange ratio during exercise [39,40,41,42,43]. However, these studies, exploratory in nature have been conducted some 25 years ago and may lack the adequate rigor, or the appropriate design. For instance, the trial conducted by Vukovich didn’t show an increase in the muscle l-carnitine content explaining possibly the lack of benefit [43]. It is also possible that the nature of the training, the length of the supplementation and the testing influence the response to the acute l-carnitine supplementation in particular. It has been reported that muscle l-carnitine content increased following l-carnitine long-term oral administration [29,44,45] whereas failed to show elevated levels after short-term supplementation [43,46]. In addition, plasma l-carnitine bioavailability does not always mirror its levels in the muscle, the driver for performance. In combination with certain mineral complexes, l-carnitine has been shown to improve aerobic exercise performance in 18–30 years old women [47].

During low to moderate exercise, long chain fatty acids represent the main source of energy. l-carnitine has been suggested to spare muscle glycogen and promote fat oxidation during exercise, and a proposed conversion of fat into energy seems likely to be reflected by a reduction in body weight [48,49,50]. In addition, l-carnitine supplementation has been shown to spare amino acid usage as a source of energy making them available potentially for new protein synthesis [51]. It was reported that raising dogs supplemented with l-carnitine experienced less protein degradation as a result of exercise [52]. These effects might explain the reported muscle mass increase in both animal studies and human trials [52,53].

## 4. Mechanisms Involved in the Effects of l-Carnitine on Recovery after Exercise

### 4.1. Effects of l-Carnitine on Muscle Injury during Exercise

Exercise-induced muscle damage and pain can both decrease quality of life and limit further training activity. In addition to its effects on exercise performance, l-carnitine has been described to help with recovery after exercise through different mechanisms. In a pilot trial, Maggini et al. addressed whether power output during a recovery period following strenuous workout can be raised by l-carnitine intake [59]. In 9 out of the 12 subjects receiving 2 g l-carnitine daily over a period of 5 days, there was a significant increase in power output after initial strenuous exercise. In contrast, single administration of l-carnitine before exhaustive cycling exercise did not improve performance during a second round of exercise after 3 h [60].

In a cross-over study, Giamberardino et al. showed that supplementation with l-carnitine alleviated pain, tenderness and release of creatine kinase—a marker of muscle injury-indicating that the nutrient was effective in reducing tissue disruption and subsequent leakage of cytosolic proteins [57]. In a series of work performed by Kraemer and colleagues, the favorable effect of l-carnitine on reducing exercise-induced hypoxia, subsequent muscle damage, and delayed onset muscle soreness (DOMS) was further substantiated [54,58,61,62]. Using magnetic resonance imaging (MRI) technique, it could be demonstrated that muscle disruption after strenuous exercise was reduced by daily intake of 2 g l-carnitine compared to placebo [58,61]. This was accompanied by a significant reduction in released cytosolic proteins such as myoglobin and creatine kinase, malondialdehyde (MDA), as well as a decrease in markers for purine metabolism such as hypoxanthine and xanthine oxidase [54,61]. In a study by Spiering et al., two different doses of l-carnitine were compared with respect to an influence on these metabolic markers and subjectively perceived muscle soreness [62]. The authors reported that supplementation with both 1 g/day and 2 g/day provided comparable benefit, thus providing additional evidence for the proposed potential of l-carnitine [62]. The group further established that supplementation with l-carnitine l-tartrate, equivalent to 2 g l-carnitine daily, over a period of 3 weeks increases the level of androgen receptors on muscle cells, thereby improving protein signaling, needed for recovery after exercise [55].

### 4.2. Effects of l-Carnitine on Blood Flow and Endothelial Function

The effects of l-carnitine on endothelial function and nitric oxide release have been demonstrated in animal studies and human clinical trials [56,63,67,68,69].

Kraemer and colleagues developed a new hypothesis, suggesting that supplementation with l-carnitine reduced structural and biochemical muscle damage and facilitated tissue repair by protecting against carnitine deficiency in the endothelial cells, thereby ameliorating blood flow and oxygen supply [67]. The new paradigm was based on early studies by Dubelaar and Hülsmann [68,69]. Here, it was demonstrated that muscle contractile force in dogs was significantly increased and accompanied by an elevated blood flow after infusion with l-carnitine and in the absence of increased muscle l-carnitine content [68]. In addition, l-carnitine prolonged the ability of endothelial cells to regulate blood flow during ischemia [69]. This pointed to a mechanism independent of muscle l-carnitine accretion and energy production. The authors hypothesized that improved force was due to an effect on the vasculature surrounding of the muscle [69]. Findings by Nuesch et al. supported this vascular effect [56]. It was shown that in athletes supplemented with 1 g of l-carnitine, plasma carnitine levels remained elevated after maximal exercise compared to a significant decrease in athletes without supplementation [56]. By investigating the flow-mediated dilation (FMD) after a high fat meal, Volek et al. further addressed the effects of l-carnitine on endothelial cell function in a cross-over study. After 3 weeks of l-carnitine supplementation, post-prandial brachial artery FMD in response to 5 min upper arm occlusion increased, whereas in the placebo arm, peak FMD decreased [63]. These results support the hypothesis that l-carnitine has a beneficial impact on vascular function through endothelial function modulation.

### 4.3. l-Carnitine as an Anti-Oxidant

One of the potential mechanisms involved in the role of l-carnitine supplementation during exercise recovery is its effect in mitigating oxidative stress during exercise. Muscle damage especially during eccentric exercise (active force generating lengthening contractions) is caused by immediate cellular and structural injury and subsequent biochemical responses during tissue repair [70,71]. Alteration of muscle fiber sarcomeres and the surrounding tissue can cause long-term dysfunction so that the recovery process can continue for up to 10 days [72]. It is also possible that local hypoxia induced by exercise can contribute to muscle injury and inflammation by decoupling between energy production (ATP from the Krebs cycle) and energy consumption in the cells. This can lead to the formation of reactive oxygen species (ROS). Ultimately, the release of intracellular components into the interstitium, and the subsequent inflammation lead to DOMS characterized by pain on movement, tenderness, as well as swelling and stiffness of the muscle [73]. Molecules such as hypoxanthine, MDA or creatine kinase resulting from sarcolemmal disruption, are involved in these events [73].

The antioxidant effects of l-carnitine upon exercise-induced oxidative stress were also reported by Parandak et al. [64]. Daily supplementation with 2 g l-carnitine over 14 days significantly increased total antioxidant capacity compared to placebo before and 24 h post-exercise, whereas markers of muscle damage and lipid peroxidation remained significantly lower in comparison to placebo [64]. Furthermore, Parthimos et al., found that post training supplementation with l-carnitine improved the total antioxidant status, which was otherwise observed in basketball players in the absence of supplementation [74].

While the vast majority of these studies were performed in young, healthy subjects, Ho and colleagues first provided experimental evidence for a favorable impact on recovery following exercise in middle-aged healthy men, with a mean age of 45 years, and women, aged 52 year average [65]. Again, a rise in stress markers during and after exercise, such as muscle soreness as perceived by the subjects, was attenuated by l-carnitine supplementation.

## 5. l-Carnitine and Aging: Old Molecule, New Uses

The aging may provide the future direction for the l-carnitine research and use. While clinical research indicated that the healthy young to middle-aged population can benefit from l-carnitine intake under physically challenging situations, effects of l-carnitine are still unknown in elderly suffering from physically fatigued conditions. The age-associated decline in skeletal muscle mass, strength, and overall activity, termed sarcopenia, is a multifactorial age-related condition. Factors such as decreased mobility, nutritional status, and mitochondrial function decline are all contributors to sarcopenia [75]. Alterations in protein metabolism and decline in protein synthesis have been reported with age progression in sarcopenic subjects [75]. While in healthy young individuals, protein metabolism is regulated by a balance of proteolytic and anabolic processes, there is a lack of an appropriate protein synthesis accompanied by a progressive degradation during sarcopenia, ultimately leading to physical frailty in older people. A recent study in the nematode *Caenorhabditis elegans* suggested specific aggregation proteins that contribute to the age-associated loss of muscle mass [76]. Another mechanism involved in the age-related muscle decline is the gradual loss of sensitivity to anabolic stimuli [77]. In addition, this decline in aged people was also attributed to the loss of type II fibers [78].

Sarcopenia is influenced by factors such as diet and physical activity [79]. A synergistic effect of meat consumption and resistance exercise has been shown to increase muscle protein synthesis [80] as well as muscle strength and endurance in elderly [81]. Moreover, supplemental protein intake can increase muscle mass and strength in elderly [82,83] and improve their physical performance [84]. However, other studies showed that protein by itself and without exercise is not effective in improving muscle mass and function in this population [85].

Growing evidence suggests that l-carnitine can positively affect muscle mass and revert the age-dependent decrease in muscle functioning. Muscle l-carnitine content has been shown to decline with age in healthy people [86]. Furthermore, aging leads to reduced transcription of OCTN2 mRNA [87], the l-carnitine transporter, indicating that tissue distribution and homeostasis of l-carnitine is hampered with advanced age. Consequently, a number of studies investigated the role of l-carnitine in the process of aging.

Malaguarnera et al. performed a clinical study with centenarians who received 2 g of l-carnitine/day or placebo over a period of 6 months and investigated the effects on physical and mental fatigue. Compared with the placebo group, supplementation resulted in improved muscle mass, reduced total fat mass, and improved walking capacity, suggesting a beneficial effect in this population [48]. These findings go along with previous research by the same group showing that, in elderly, body fat mass was reduced, whereas muscle mass increased. This was accompanied by a significant reduction in physical and mental fatigue [49]. Supplementation with acetyl-l-carnitine, the acetylated derivative of l-carnitine, also decreased physical and mental fatigue in subjects 70 year old [50]. In a double-blind, randomized, placebo-controlled clinical trial with pre-frail older subjects with a mean age of 68 years, Badrasawi et al. demonstrated a significant improvement on frailty status after 10-week supplementation with 1.5 g l-carnitine per day [88]. A recent study by Evans et al. provided evidence that a combination of l-carnitine, creatine, and leucine favorably affects muscle mass and performance [53]. In this randomized placebo-controlled double blind study in subjects aged 55–70 years, researchers investigated the potential synergistic effect of this novel formulation in comparison to placebo after eight-week supplementation. It was found that subjects significantly improved a composite outcome measure of body mass, muscle strength and a 6-min walk test, compared to placebo, and increased total lean muscle mass, leg lean muscle mass, and leg strength. l-carnitine supplementation alone did not show a significant improvement in the composite parameter compared to placebo, yet, subjects maintained both the composite score and their leg muscle strength compared to baseline, whereas, in the placebo group, both declined during the course of the study [53].

Although, in the younger population, a number of studies could not show weight loss after intake of l-carnitine [89], in a recent meta-analysis, Pooyandjoo and colleagues came to the conclusion that body weight was significantly reduced in subjects who received l-carnitine compared with the control groups. The studies that were included in the meta-analysis were mostly performed in obese and/or diabetic subjects [90].

Although the mechanisms underlying the effects of l-carnitine in promoting muscle mass and function in older people are still unknown, some of the general mechanisms of action of l-carnitine effects shown in animal studies and younger athletes can apply. By converting fat into energy, supplemental l-carnitine allowed for sparing of amino acids thereby leading to protein accretion in the muscle of swine [51]. Keller and colleagues demonstrated a role of l-carnitine in the transcriptional regulation of genes involved in the ubiquitin proteasome system of the skeletal muscle of piglets, indicating a potential mechanism by which l-carnitine prevents muscle protein degradation [91]. In addition, l-carnitine was reported to increase IGF-1 and Akt, thereby inducing the mammalian target of rapamycin (mTOR) signaling pathway, which is a key modulator of protein anabolism [92].

As postulated by the free radical theory of aging, peroxidative damage promotes the process of aging [93]. Antioxidants can scavenge reactive oxygen species or prevent their production thereby alleviating the oxidative stress. The antioxidant properties of l-carnitine have been indicated by several clinical studies [64,74,94], suggesting a potential additional mode of action by which l-carnitine may impede the biochemical mechanisms underlying tissue aging.

Another hallmark of aging is accelerated neuronal cell injury and death leading to brain shrinkage and a decline in the brain function. It was suggested that mitochondrial decay may be the predominant underlying event [95]. Recently, Nicassio and colleagues suggested an influence of acetyl-l-carnitine on mitochondria homeostasis in the brains of old rats [96].

## 6. Conclusions

As a key player in fatty acid metabolism and energy production, the role of l-carnitine in diverse indications has been matter of scientific investigations. l-carnitine has been used as an ergogenic aid for professional athletes and as a dietary supplement in the physically active population. An abundance of human studies performed in healthy active subjects, resistance-trained athletes, or untrained men and women examined the effect of the nutritional supplement on physical performance, oxygen capacity, or muscle strength. More recently, clinical research shifted to evaluating the hypothesis that l-carnitine intake facilitates the recovery process after exercise. Scientific data indicates that the athletic population can benefit from l-carnitine intake as it attenuates the side effects of high-intensity training by reducing the magnitude of exercise-induced hypoxia and muscle injury. 

Under healthy conditions and in the absence of stress, l-carnitine availability is not a limiting factor in fatty acid β-oxidation. Its homeostasis is highly regulated by bioavailability, transport, and urinary excretion. Yet, under conditions with aberrations, such as inborn or acquired carnitine deficiency, hemodialysis, or in sarcopenic and frail subjects, supplementation with l-carnitine has been shown to increase physical performance and muscle mass and function. The age-related muscle decline can be reversed by both physical activity and nutritional means. While endurance exercise can be arduous in elderly, nutritional supplementation in conjunction with moderate exercise may be a potential strategy to slow down sarcopenia, as one hallmark of frailty, in older adults.

A growing number of animal studies provided evidence for the multifaceted mechanisms, by which l-carnitine exerts its beneficial action on increased protein synthesis and reduced muscle degradation. In addition, regulation of mitochondrial homeostasis by l-carnitine during aging is postulated to impact the age-related declines. Thus, dietary supplementation with l-carnitine may contribute to help during the aging process by impeding the progression of muscle mass and function decline as well as neurodegeneration. Further research in warranted in this area.

In conclusion, given the impact of the structural and symptomatic consequences of high-intensity exercise, i.e., impairment of muscle sarcomeres and pain, which do not only reduce quality of life but diminish further training capacities, facilitation of recovery from exercise by l-carnitine supplementation is particularly beneficial to the healthy young active population. Moreover, elderly experiencing lean muscle mass and function decline, reduced muscle l-carnitine content and mitochondrial dysfunction can also benefit from the positive impact of supplementation with l-carnitine. In particular, with a growing number of older subjects engaged in moderate exercise, the role of l-carnitine in this demographic will continue to gain importance. 

## Figures and Tables

**Table 1:** Summary of clinical studies investigating the effect of l-carnitine on exercise performance and recovery.

Authors/Title	# Subject	Age (Mean or Range)	Dose Duration	Outcome
**Athletes/Well Trained (Professionals, Age 16–36)**				
**[29]** Carnitine in muscle, serum, and urine of nonprofessional athletes: effects of physical exercise, training, and l-carnitine administration.	24 athletes	19–27	1 g BID for 6 mo of training	Prevention of training decreased total and free. Carnitine, positive effect on recovery.
**[33]** Studies concerning the ergogenic value of protein supply and l-carnitine in elite junior cyclists.	7 junior athletes	na	1 g/d for 6 wk and 2 g/d for 10 d (before competition)	Supplemented group showed better stress-induced efforts and obtained higher performances.
**[34]** Studies concerning chronic and acute effects of l-carnitine in elite athletes.	110 athletes (in 6 studies)	16–33	4 g oral of 1 g iv (single dose) 3 g/d for 3 wk or placebo	Single dose: beneficial effects on physical output, lipid metabolism, muscular function (contraction), lactate accumulation after exercise and urine mucoproteins. 3 week treatment: Beneficial effects on the lipid metabolism, evoked muscular potential, VO_2_max, behavior and the biological output.
**[35]** Respiratory chain enzymes in muscle of endurance athletes: effect of l-carnitine.	14 athletes	na	2 g BID for 4 wk of training	Increase in respiratory-chain enzyme activities in the muscle.
**[36]** The effects of acute l-carnitine supplementation on endurance performance of athletes.	26 athletes	18.42 ± 0.5	12 received 3 g 14 received 4 g	Compared to placebo, l-carnitine supplemented groups showed lower lactate levels and lower heart rate.
**[40]** Effects of l-carnitine supplementation on physical performance and energy metabolism of endurance-trained athletes: a double-blind crossover field study.	7 athletes	36 ± 3	2 g before start and after 20 km run	Significant increase in l-carnitine plasma concentration. No effect on performance or metabolism.
**[54]** Effects of l-carnitine l-tartrate supplementation on muscle oxygenation responses to resistance exercise.	9 healthy, previously resistance trained men	25.2 ± 6	2 g/d for 23 d or placebo	Enhanced oxygen consumption => hypoxic stress is attenuated with carnitine supplementation.
**[55]** Androgenic responses to resistance exercise: effects of feeding and l-carnitine.	10 resistance-trained men	22 ± 1	2 g/d for 21 d or placebo	Increased androgen receptor content and enhanced luteinizing hormone.
**[56]** Plasma and urine carnitine concentrations in well-trained athletes at rest and after exercise. Influence of l-carnitine intake.	9 athletes	na	1 g before and after treadmill ergometer or placebo	No decrease in serum carnitine levels after exercise in the supplementation group. No effect on maximal exercise. No effect on maximal exercise.
**[32]** Effects of exercise intensity and altered substrate availability on cardiovascular and metabolic responses to exercise after oral carnitine supplementation in athletes.	15 athletes	Pl: 31 ± 8 LC: 34 ± 10	3 g/d for 15 d or placebo	No significant difference between whole-body rates of CHO and fat oxidation at any workload. At day 15, heart rate and blood glucose concentration were lower during exercise in the l-carnitine group compared to Placebo.
**[31]** Carbohydrate, protein, and fat metabolism during exercise after oral carnitine supplementation in humans.	20 active male athletes	Pl: 32 ± 9 LC: 34 ± 10	2 g/d for 2 wks or placebo	After 2 wk of l-carnitine supplementation, plasma ammonia response to exercise tended to be suppressed. No effects on fat, carbohydrate, or protein contribution to metabolism during prolonged moderate-intensity cycling exercise
**Healthy (recreationally active, age 18–50)**				
**[37]** Metabolic changes induced by maximal exercise in human subjects following l-carnitine administration.	10 moderately trained men	18.42 ± 0.50	2 g before high-intensity exercise	Stimulation of PDH activity, and decrease in plasma lactate and pyruvate.
**[38]** Influence of l-carnitine administration on maximal physical exercise.	10 moderately trained men	22–30	2 g before high-intensity exercise	Increased VO_2_max.
**[39]** Effects of four weeks l-carnitine l-tartrate ingestion on substrate utilization during prolonged exercise.	15 trained males	20–46	3 g for 4 wk or placebo	No effect on substrate utilization or endurance performance.
**[57]** Effects of prolonged l-carnitine administration on delayed muscle pain and CK release after eccentric effort.	6 untrained subjects	26 ± 3.8	3 g/d for 3 wk	Protective effect against pain and damage from eccentric effort.
**[58]** The effects of l-carnitine l-tartrate supplementation on hormonal responses to resistance exercise and recovery.	10 healthy, recreationally weight-trained men	23.7 ± 2.3	2 g/d for 3 wk	Increased IGFBP-3 concentrations prior to and at 30, 120, and 180 min after acute exercise => protection from muscle damage.
**[41]** Effect of l-carnitine on submaximal exercise metabolism after depletion of muscle glycogen.	9 healthy males	24.9 ± 1.0	3 g/d for 7 d	No effects on fat oxidation, RQ, perceived exertion, lactate, heart rate during exercise after glycogen depletion.
**[42]** The effects of l-carnitine supplementation on performance during interval swimming.	20 (swimmers)	20.1 ± 0.6	2 g BID for 7 d or placebo	Elevation in serum l-carnitine and carnitine fractions. No differences in performance times between trials or groups was observed; similar response related to blood pH, LA and BE in both groups during each trial was revealed.
**[43]** Carnitine supplementation: effect on muscle carnitine and glycogen content during exercise.	8	26.8 ± 2.31	4 g/d for 14 d	Increase in serum carnitine. No effect on muscle carnitine content, lipid oxidation and lactate accumulation.
**[59]** l-carnitine supplementation results in improved recovery after strenuous exercise—a preliminary study.	12 (trained/untrained)	25.7 ± 4	2 g/d for 5 d	Improved recovery in 9 of 12 subjects.
**[60]** l-carnitine and the recovery from exhaustive endurance exercise: a randomised, double-blind, placebo-controlled trial.	12	25 ± 3	2 g/d for 14 d or placebo	2 g of l-carnitine taken 2 h before a first of 2 constant-load exercise tests had no influence on the second tests performed 3h after the first test compared with placebo.
**[61]** l-Carnitine l-tartrate supplementation favorably affects markers of recovery from exercise stress.	30 healthy subjects	30 ± 8	2 g for 3 wk or placebo	Improvement in postprandial vascular functions after a high-fat meal.
**[62]** Responses of criterion variables to different supplemental doses of l-carnitine l-tartrate.	8 healthy men	22 ± 3	0, 1 g, 2 g for 3 wk	Increase in serum carnitine concentrations. Reduction in post-exercise serum hypoxanthine, serum xanthine oxidase, serum myoglobin, and perceived muscle soreness. Reduced metabolic stress, less muscle damage.
**[63]** Effects of carnitine supplementation on flow-mediated dilation and vascular inflammatory responses to a high-fat meal in healthy young adults.	30 healthy men and women	30 ± 8	2 g/d for 3 wk or placebo	Improvement in postprandial flow-mediated dilatation after a high-fat meal.
**[64]** The effect of two-week l-carnitine supplementation on exercise-induced oxidative stress and muscle damage.	21 active healthy men	About 22	2 g/d for 14 d or placebo	Increase in total antioxidant capacity after 14d and 24h post exercise. Lower malondialdehyde-TBARS, creatine kinase and lactate dehydrogenase 24 h post exercise.
**[65]** l-Carnitine l-tartrate supplementation favorably affects biochemical markers of recovery from physical exertion in middle-aged men and women.	18 healthy men and women	m: 45.4 ± 5.3 f: 51.9 ± 5.0	2 g/d for 24 d	Positive effects on purine metabolism, free radical formation, muscle tissue disruption, muscle soreness. No effect on physical performance.
**[30]** Long-term administration of l-carnitine to humans: effect on skeletal muscle carnitine content and physical performance.	8 healthy male adults	23–25	2 × 2 g/d for 3 months	No significant differences between V_O2_max, RER_max_, and P_max_ between the three time points investigated: pre/post at baseline and post exercise after 3 months. No pre/post difference in muscle carnitine content at baseline and post-exercise at 3 months. Activities of citrate synthase and cytochrome oxidase, as well as the skeletal muscle fiber composition remained unaffected.
**[66]** Prolonged submaximal exercise and l-carnitine in humans.	10 young males	na	2 g/d for 4 wks; followed by 0 g/d for 6–8 wks	Twenty-five percent increase in free and total L-carnitine plasma levels during supplementation. These levels returned to normal 6–8 weeks after the supplementation stopped. No changes in endogenous lipids for fuel supply, indicating possibly that this population has sufficient levels of L-carnitine
**Elderly (age 55–106)**				
**[48]** l-Carnitine treatment reduces severity of physical and mental fatigue and increases cognitive functions in centenarians: a randomized and controlled clinical trial.	66 centenarians	100–106	2 g/d or placebo for 6 mo	Reduction of total fat mass, increases total muscular mass, and facilitates an increased capacity for physical and cognitive activity by reducing fatigue and improving cognitive functions.
**[49]** Levocarnitine administration in elderly subjects with rapid muscle fatigue: effect on body composition, lipid profile and fatigue.	84 elderly subjects	81.5 ± 6.7	2 g BID for 30 day or placebo	Improvements in the following parameters: total fat mass, total muscle mass, total cholesterol, LDL-C, HDL-C, triglycerides, apoAl, and apoB. Decreased physical and fatigue.
**[50]** Acetyl l-carnitine (ALC) treatment in elderly patients with fatigue.	96 aged subjects	71–88	2 g BID for 180 d or placebo	Reduction in both physical and mental fatigue and improvement of both the cognitive status and physical functions.
**[53]** Efficacy of a novel formulation of l-Carnitine, creatine, and leucine on lean body mass and functional muscle strength in healthy older adults: a randomized, double-blind placebo-controlled study.	42 healthy older adults	55–70	1.5 g carnitine or carnitine combination or placebo for 8 wk	l-Carnitine combined with creatine, l-leucine, and Vitamin D significantly improved muscle mass and strength compared to placebo; increase in mTOR protein level.

Abbreviation used: BID: twice per day; d: day; wk: week; mo: month; CHO: carbohydrates; Pl: placebo; LC: l-Carnitine; PDH: pyruvate dehydrogenase; VO_2_max: maximal oxygen uptake; CK: creatine kinase; IGFBP-3: insulin-like-growth-factor-binding-protein-3; RQ: respiratory quotient; LA: lactate; BE: base excess; TBARS: thiobarbituric acid reactive substances; RER_max_: maximal respiratory exchange ratio; P_max_: maximal power; LDL-C: low density lipoprotein cholesterol; HDL-C: high density lipoprotein cholesterol; apoA1: Apolipoprotein A1; apoB: apolipoprotein B; mTOR: mechanistic target of rapamycin.
