# Erratum: l-Carnitine Supplementation in Recovery after Exercise; *Nutrients* 2018, *10*, 349

**DOI:** 10.3390/nu10050541

**Published:** 2018-04-26

**Authors:** Nutrients Editorial Office

**Affiliations:** MDPI, St. Alban-Anlage 66, 4052 Basel, Switzerland; nutrients@mdpi.com

The *Nutrients* Editorial Office would like to report errors in the published paper [1]. The details are as follows:

(1) In Table 1 the subheading on page 7 and 8 should be changed to “Healthy (recreationally active, age 18–50)”.
(2) In Table 1 on page 8, the following content should be added into the outcome column for Reference 66: “Twenty-five percent increase in free and total l-carnitine plasma levels during supplementation. These levels returned to normal 6–8 weeks after the supplementation stopped. There were no changes in endogenous lipids for fuel supply, indicating the possiblity that this population has sufficient levels of l-carnitine.”

We apologize for any inconvenience caused to the readers by this change. The change does not affect the scientific results. The manuscript will be updated and the original will remain available on the article webpage.
